# Verrucous carcinoma of the oral mucosa: An epidemiological and follow-up study of patients treated with surgery in 5 last years

**DOI:** 10.4317/medoral.19683

**Published:** 2014-06-01

**Authors:** Alberto Candau-Alvarez, Alicia Dean-Ferrer, Francisco J. Alamillos-Granados, Susana Heredero-Jung, Blas García-García, Juan J. Ruiz-Masera, Rafael Arévalo-Arévalo, Francisco Zafra-Camacho, Borja Valenzuela-Salas

**Affiliations:** 1 Resident. Oral and Maxillofacial Surgery Department. Hospital Universitario “Reina Sofía”, Córdoba (Spain); 2 Head of Department. Oral and Maxillofacial Surgery Department. Hospital Universitario “Reina Sofía”, Córdoba (Spain); 3 Consultant. Oral and Maxillofacial Surgery Department. Hospital Universitario “Reina Sofía”, Córdoba (Spain)

## Abstract

Introduction: Oral Verrucous Carcinoma (OVC) is described apart of the Squamous Cell Carcinoma (SCC) due to its specific properties. The objective of our study is to show our series of cases of OVC and to compare with the SCC in terms of clinical manifestations, epidemiology, histopathology, treatment and follow-up. Material and Methods: This is a retrospective study of all the OVC treated in our department between January-2007 and December-2011. The analyzed variables were sex, age, localization in the oral cavity, histopathology, number of biopsies needed to diagnose OVC, TNM classification, treatment and recurrences during follow-up. Results: Our sample was composed by n=14 patients, 57% female, with a mean age of 69.14 years. The most common localization was buccal mucosa (n=5). Seven patients were diagnosed of OVC with the first biopsy. TNM classification was: pT1: 7 patients, pT2: 3 patients, pT3: 3 patients, pT4: 1 patient. No cervical metastases were observed either in cervical neck dissection or during the follow-up of the patients. The treatment was surgery with clinical resection margins up to 1 cm in all cases, followed by radiotherapy in selected cases. Only n=1 patient (7.69%) presented a recurrence after 34 months of follow-up. The overall survival rate was 92.85%. Conclusions: In our population, OVC represents the 6.16% of all oral cavity and oropharynx cancer, and is more frequent in female patients above 70 years old. It uses to rise over a previous lesion, and usually affects the buccal mucosa. In patients with high suspicious lesions, more than one biopsy may be needed to diagnose OVC. No patient showed cervical dissemination. In our experience, treatment based on local resection, without cervical neck dissection, could be a good option for these patients.

** Key words:**Verrucous carcinoma, squamous cell carcinoma, oral cancer, oral cavity, epidemiology, follow-up.

## Introduction

The Verrucous Carcinoma was described by LV Ackerman in 1948 (1) as an infrequent subtype of malignant disease which affects oral cavity. Usually, OVC presents a high tendency of local invasion, with a low tendency of dissemination, which varies depending on tumour size and evolution time (2), with a very low tendency to metastasize. Previous lesions as leucoplakias or eritroplakias, as well as Proliferative Verrucous Leucoplakia, are the sites where the OVC uses to arise from (3-5).

Its etiology is not well known, but smoking habit, alcohol consumption and betel nut chewing are proved causes. The role of the Human Papilomavirus in OVC oncogenesis is much less important than in the SCC oncogenesis (6-8).

Verrucous carcinoma usually debuts as an abnormal growth or as change in the consistency of a previous potentially malignant disorder of the oral cavity. All mucosal sites of the oral cavity can be affected. However, the rate of malignant transformation of a leukoplakia to an OVC is 20.81 times higher if they are located in the gingiva in comparison with the tongue (9).

**Figure 1:**
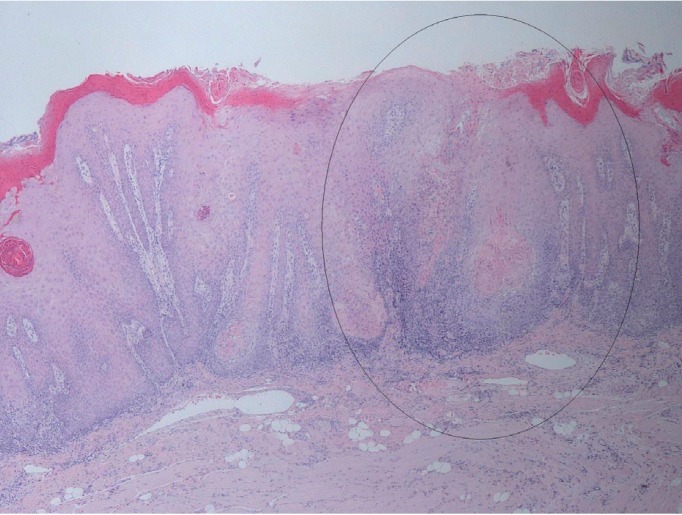
General histopathological characteristics of the excised specimens revealed acanthosis, papillomatosis, and hyperkeratosis of the epithelium of the lesion, continuing with characteristics of healthy mucosa. Squamous epithelial cell composition of the tumors did not give a definite atypical character, but showed blunt rete processes toward the subepithelial area. Lymphocyte infiltration was noted in the periphery of the tumor islands. Hematoxylin-Eosin stain (H-E) 2x.

Histopathologically, it frequently shows aneuploidy (10), which can be shown in conventional exfoliative cytology biopsies, which can be used as a marker of progression from potentially malignant disorder of the oral cavity to an OVC (Fig. 1).

Surgery is the best treatment. Although an optimal disease control can be achieved by surgery only, frequent revision is mandatory due to the increased risk of second tumours (11).

Our objective is to show our series of OVC patients, treated at our department, and to compare with the SCC in terms of clinical manifestations, epidemiology, histopathology, treatment and follow-up.

## Material and Methods

We designed a descriptive and retrospective epidemiological study of patients diagnosed of OVC and treated by surgery in our institution between January/2007 and December/2011.

Between those years, we collected a total of 14 OVC patients, among n=227 patients diagnosed of SCC of the oral cavity, which met the following Inclusion Criteria: pathological confirmation of OVC of the biopsied specimen of the resected tumor, surgical treatment and follow-up during more than 6 months. We excluded all patients who did not fit the inclusion criteria.

The analyzed variables were: sex, age, localization in the oral cavity, histopathology, number of biopsies needed to diagnose OVC, TNM classification (based on American Joint Committee on Cancer, 7th edition), treatment given and recurrences during follow-up. The statistical descriptive analysis was done using the SPSS v17.0.

## Results

In our sample, 57% (n=8) were woman, and 43% (n=6) were males. The mean age was 69.14 years (range 84-46). The most frequent localization was buccal mucosa (n=5 patients), followed by lipcommissure (n=3), gingiva (n=3), tongue (n=2) and hard palate (n=1). In n=2 patients histopathological study revealed “hybrid verrucous carcinoma”. More epidemiological data are shown in  Table 1. Half of the patients (n=7) needed more than one biopsy to establish OVC diagnosis. Those previous biopsies were informed as epithelial hyperplasia (n=3), parakeratosis with acantosis (n=2), simple keratosis (n=2) and verrucous hyperplasia (n=1). The TNM classification was pT1 (n=7), pT2 (n=3), pT3 (n=3), and pT4 (n=1). The treatment in all cases was local resection with clinical margins above 1cm. In n=3 cases, a supraomohyoid cervical neck dissection were performed, and a radical neck dissection in n=1 case. The mean histopathological distance to deep resection margin was 5.35mm (median 5.5mm). Radiotherapy was administered to T3 and T4 patients and in patient #4 due to close histopathological margin (1 mm). The mean follow-up was 24.76 months (range 6-53 months). Data about surgery and follow-up are shown in  Table 2. Only n=1 patient (#6) died due a massive hemorrhage in his early postoperatory period, and was not considered to perform follow-up and recurrence rate statistics. The recurrence rate was 7.69% (n=1/13 patients), which occurred after 34 months. The overall survival rate was 92.85% (13/14 patients).

**Table 1:**
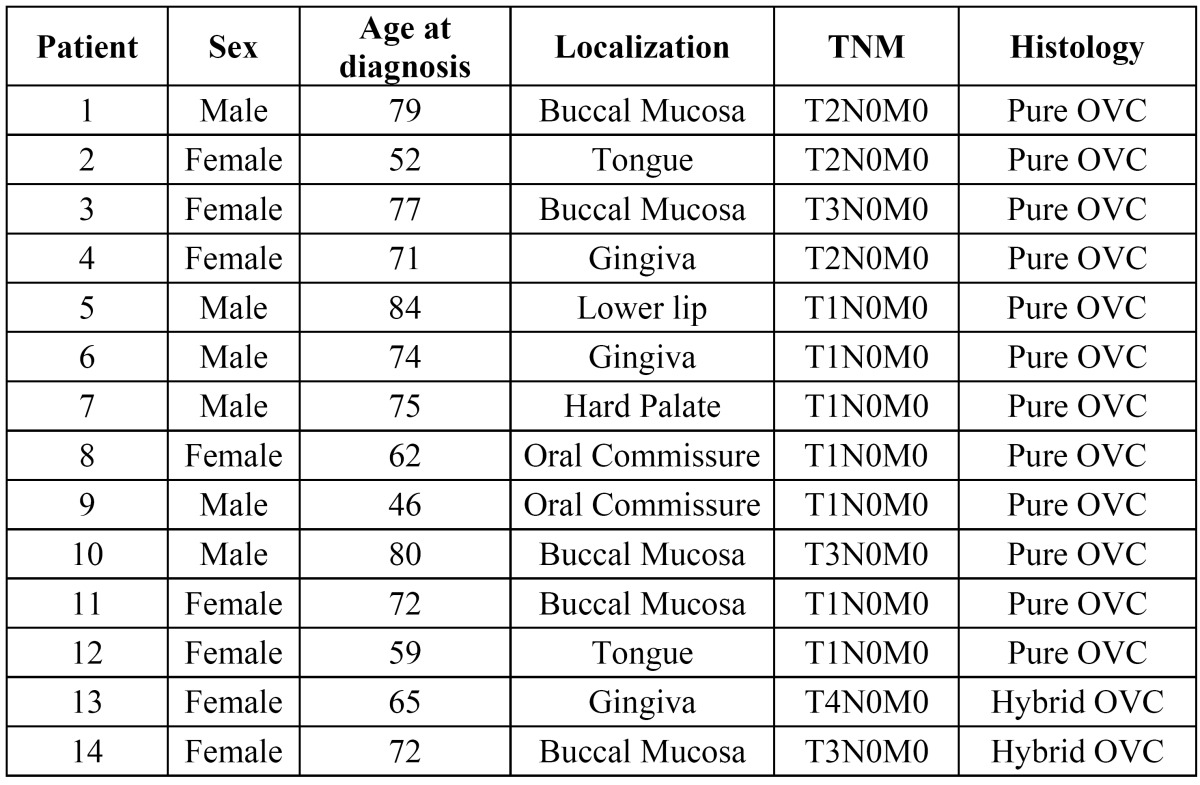
Epidemiological description of the Oral Verrucous Carcinoma.

**Table 2:**
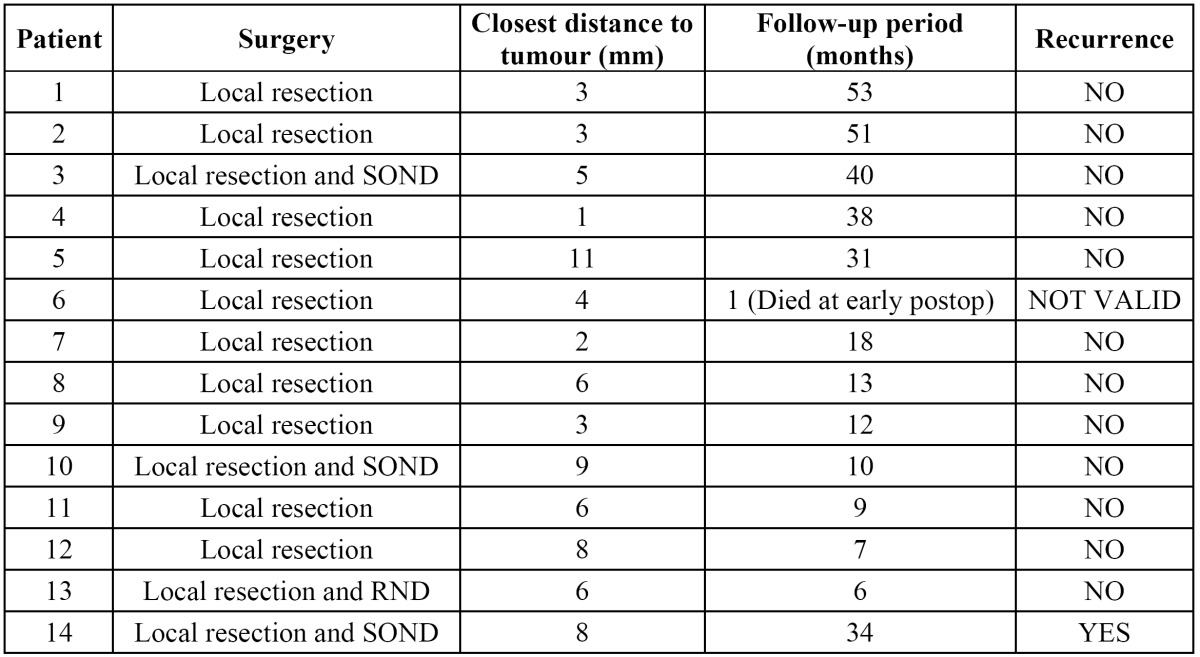
Treatment and Follow-Up of the Oral Verrucous Carcinoma patients. SOND: Supra-Omohyoid Neck Dissection. RND: Radical Neck Dissection.

## Discussion

The prevalence of OVC for total carcinomas affecting the oral cavity and oropharynx is low, and as revised in Rekha *et al.* (12), is between 2-12%. In our series, verrucous carcinoma of the oral cavity, with respect to all patients with oral cavity cancer, represents the 6.16%, being in consonance with the reviewed literature.

Published series on Asian patients, reflect its greater incidence in men with prevalence as high as 77.4% (13) and 94.9% (14). This seems to be explained by consumer culture of betel nut and tobacco chewing. To the best of our known, no previous studies reflects the gender tendency of OVC in Spanish Caucasian population. In our sample, 57% were women, which can be in consonant with a population without tobacco chewing habits. Numerous studies (12-15) indicate that OVC primarily affects patients between 40 and 60. The mean age at diagnosis in our patients was 69.14 years on average (median 71.5 years), which is almost 10-15 years later in our series than in the literature. The earlier affectation in these patients may be due, in the opinion of the authors, to an early onset in the consumption of carcinogens such as betel nut.

**Figure 2:**
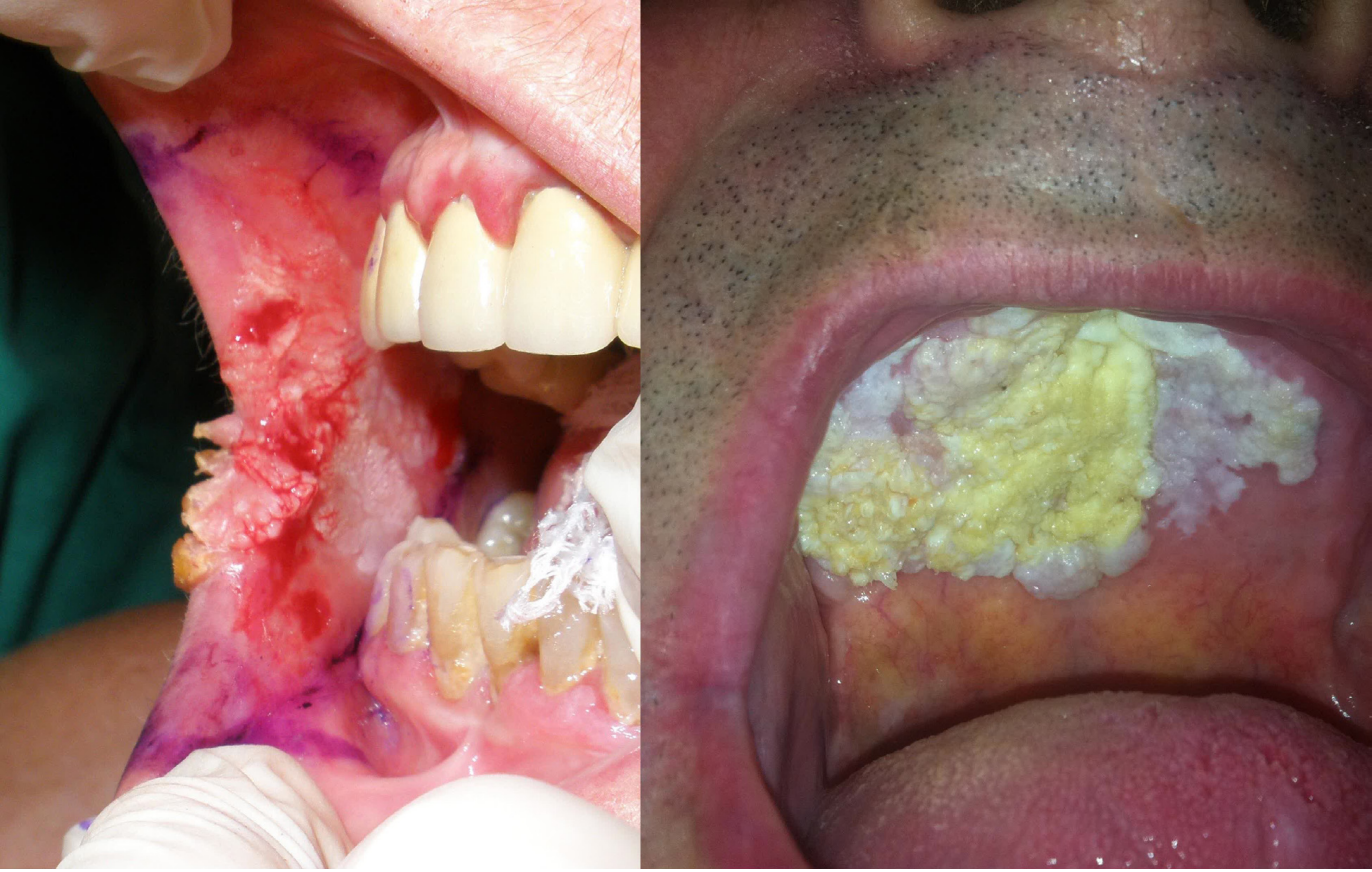
Different localizations of OVC. Left: Buccal mucosa (patient #11). Right: Hard Palate (patient #7).

The involvement of buccal mucosa appears in several articles as the most common (12-14), as well as in our study (Fig. 2). Other authors have reported a greater involvement of the lower lip (15,16), alveolar ridge and gingiva (3,17). The presence of a previous leukoplakia is also a constant (2-5,12,13). Some authors report diagnoses like verrucous leukoplakia (17) or verrucous hyperplasia (18) in biopsies of long standing leukoplakia in which an OVC appeared later. In our study we found n = 7 OVC patients with previous verrucous leukoplakia in which subsequent biopsies were performed, with histopathological diagnoses similar to those described before. All our patients were treated by surgery. Local resection with 1 centimeter of clinical margin is considered by many authors (19,20) as the treatment of choice for verrucous carcinoma. Although clinical margins were even greater than 1 cm, due to tissue retraction, histological margins were closer to the tumor. However, histological margins above 5 mm were considered sufficient to not increase the risk of local recurrence (21). In patient #4, due to its closest free margin (1 mm) and the impossibility to increase resection margins in a second surgery, received postoperative radiotherapy.

The need for a cervical dissection is also controversial. Case series published over the years agree that the OVC tends to grow locally but not to cause ganglionar spread (22,23). However, there are few published papers that refer of distant metastases as in the orbit (24) and axillary lymph nodes (25). Thereby, cervical dissection should not be indicated in all cases of OVC. Because of an initial biopsy of SCC in patients #3 and #10, a Supraomohyoid Neck Dissection was performed due to the size of the tumor (T3 and T4), which were informed as negative (pN0) in both cases. Also T3 and T4 patients received radiotherapy after surgery.

We have identified an important diagnostic problem, which is the differentiation between “pure” OVC and “hybrid” OVC, which has foci of squamous cell carcinoma. The hybrid OVC can be up to 20% of diagnosed OVC, and behaves like squamous cell carcinomas regarding to their metastatic spreading tendency (26). In these cases, performing a cervical neck dissection is recommended, as if it were a squamous cell carcinoma. In our series, in n=2 patients (patients #13 and #14), the histopathological study was informed as hybrid OVC, while the rest were pure OVC. One of these 2 patients (patient #13) was diagnosed preoperatively of hybrid OVC, and due both to this diagnosis and to the size of the tumor (T4), underwent a Modified Radical Neck Dissection type III (preserving the Internal Jugular Vein, the Spinal Nerve and the Sternocleidomastoid Muscle) which was informed as negative (pN0). The other patient (case #14) had an initial biopsy of OVC, and underwent local wide resection, and after the histopathological study was reclassified as a hybrid OVC, and secondary performed a Supraomohyoid Neck Dissection which was informed as negative (pN0). Neither cases of hybrid OVC exhibited metastasis along the follow-up (6 and 34 months, respectively). Also, as befits a pure OVC (20), no patient had cervical lymph node metastases, either in previous imaging studies, or in the histopathological studies in those who underwent prophylactic neck dissection.

During the follow-up of our patients, only one patient had a local tumor recurrence 34 months after surgery in the buccal mucosa, biopsied as OVC again, and treated by surgery. No second tumors were found. Since the follow-up period was uneven for all patients, we can not state the recurrence rate at 5 years. Because the average follow-up was close to 2 years, we can say that the recurrence rate at 2 years of follow-up stood at 7.69%. The long-term follow-up studies of OVC patients refer highly variable recurrence rates between 0 and 66.7% at 5 years (13,15,19), which mostly depend on the type of treatment that has been given. Overall survival of the series was 92.85% (13/14 cases) for the follow-up period described above. Survival of patients with OVC to 5 years is between 93.65% (20) and 94.7% (19).

The most relevant limitation found in this article is the small size of the registered sample. Because our institution gives assistance to a population of a small city, the volume of oncological patients treated could be smaller than those that appear in other published series. Although n=14 patients represent a small sample, the prevalence of OVC among all SCC or the oral cavity in our series is similar to those published and referenced above. That is why we consider our sample as representative.

## Conclusion

Verrucous carcinoma in our series appears in patients around 70 years, usually on a previous injury, often affecting the buccal mucosa. In patients with suspicious lesions more than one biopsy to diagnose a verrucous carcinoma may be needed. No patient had cervical lymph node involvement. Local resection with at least 5 mm of histological margin, without prophylactic neck dissection allows local control of the disease with recurrence rates of 7.69% at 2 years. Overall survival rate in our series was 92.85%.
